# Immediate and Long-Term Effects of Hyperbaric Oxygenation in Patients with Long COVID-19 Syndrome Using SF-36 Survey and VAS Score: A Clinical Pilot Study

**DOI:** 10.3390/jcm12196253

**Published:** 2023-09-28

**Authors:** Joerg Lindenmann, Christian Porubsky, Lucija Okresa, Huberta Klemen, Iurii Mykoliuk, Andrej Roj, Amir Koutp, Eveline Kink, Florian Iberer, Gabor Kovacs, Robert Krause, Josef Smolle, Freyja Maria Smolle-Juettner

**Affiliations:** 1 Division of Thoracic Surgery and Hyperbaric Surgery, Department of Surgery, Medical University of Graz, 8036 Graz, Austriafreyja.smolle@medunigraz.at (F.M.S.-J.); 2 Department of Internal and Respiratory Medicine, Hospital Graz II, Academic Teaching Hospital of the Medical University of Graz, 8036 Graz, Austria; 3 Ludwig Boltzmann Institute for Lung Vascular Research, Medical University of Graz, 8036 Graz, Austria; 4 Division of Pulmonology, Department of Internal Medicine, Medical University of Graz, 8036 Graz, Austria; 5 Division of Infectious Diseases, Department of Internal Medicine, Medical University of Graz, 8036 Graz, Austria; 6 Institute of Medical Informatics, Statistics and Documentation, Medical University of Graz, 8036 Graz, Austria

**Keywords:** long COVID, hyperbaric oxygenation, long-term effect, improvement, physical function, pain, fatigue, energy, general perception of health

## Abstract

(1) Background: Long COVID syndrome (LCS) is a heterogeneous long-standing condition following COVID-19 infection. Treatment options are limited to symptomatic measures, and no specific medication has been established. Hyperbaric oxygenation (HBO) has been found to have a positive impact on the treatment of COVID-19 infection. This study evaluates both the feasibility and outcome of supportive HBO in patients with LCS. (2) Methods: Within 17 months, 70 patients with proven LCS were prospectively included. Each patient underwent a cycle of 10 subsequent HBO treatment sessions administered for 75 min at 2.2 atmospheres. Evaluation of the patients was performed before the first and after the last HBO session and 3 months afterwards. Statistical evaluation was based on an intention-to-treat analysis using Fisher’s exact test and Student’s *t*-test for paired samples. (3) Results: In total, 59 patients (33 females, 26 males; mean age: 43.9 years; range: 23–74 years; median: 45.0) were evaluable. After HBO, a statistically significant improvement of physical functioning (*p* < 0.001), physical role (*p* = 0.01), energy (*p* < 0.001), emotional well-being (*p* < 0.001), social functioning (*p* < 0.001), pain (*p* = 0.01) and reduced limitation of activities (*p* < 0.001) was confirmed. (4) Conclusions: Physical functioning and both the physical and emotional role improved significantly and sustainably, suggesting HBO as a promising supportive therapeutic tool for the treatment of LCS.

## 1. Introduction

### 1.1. Background

Long COVID syndrome (LCS), also called post-COVID-19 syndrome or long-haul coronavirus disease 2019 (COVID-19) [1], is a heterogenous condition affecting patients following COVID-19 infections.

Though a variety of defining criteria have been proposed, the most common description is symptoms extending for more than 12 weeks beyond the initial COVID-19 infection [1]. Among individuals with LCS 3 months after symptomatic severe acute respiratory syndrome coronavirus type 2 (SARS-CoV-2) infection, an estimated 15.1% continued to experience symptoms at 12 months [2].

LCS evolves either directly from the initial COVID-19 infection or develops after a symptom-free interval and affects patients regardless of the severity of the COVID-19 disease. Though LCS is predominantly found in younger, female individuals, it relates to all age groups and both sexes. The array of symptoms varies individually and derives from three LCS symptom clusters: (a) persistent fatigue, mood swings, body pain; (b) cognitive impairment; and (c) ongoing respiratory problems [2]. The dimension of the problem is considerable. Wulf-Hanson et al. reviewed reports of 1.2 million formerly symptomatic COVID-19 patients. They estimated that at least 6.2% of them experienced at least one out of the three LCS symptom clusters [2].

Physical symptoms relating to the cardiorespiratory system are common and, in many cases, they persist despite normal objective parameters [2]. In addition, mental disorders, headaches, smell and taste dysfunction, hair loss, insomnia, rhinorrhea and gastrointestinal issues are frequently experienced in LCS. In some cases, the condition is highly debilitating, resulting in an inability to return to work or even perform household chores [3], not only because of the physical symptoms but also due to the psychic impact of LCS [4].

Treatment options are currently limited to symptomatic measures including physical rehabilitation, and support by mental and social services alongside monitoring of symptoms. Since there is insufficient understanding of the mechanisms underlying LCS, no specific medication has been established yet [3,5].

Hyperbaric oxygenation (HBO) denotes the inhalation of 100% oxygen at pressures exceeding one atmosphere absolute, thus enhancing the amount of oxygen dissolved in the body tissues. During HBO, the arterial oxygen (O_2_) tension typically exceeds 100 mmHg.

Depending on the pressure applied, arterial O_2_ tension reaches 1300 to 2000 mmHg, equaling 200–400 mmHg in tissues. Even though many of the beneficial effects of HBO can be explained by the improvement of tissue oxygenation, it is now understood that the combined action of hyperoxia and hyperbaric pressure triggers both oxygen- and pressure-sensitive genes, resulting in inducing regenerative processes. These include stem cell proliferation and mobilization, enhancement of anti-apoptotic and anti-inflammatory factors, and downregulation of inflammatory cascades [6].

For acute COVID-19 infection, there are anecdotal reports about compassionate use [7,8] and first prospective studies [9,10] of HBO, showing accelerated improvement of hypoxemia but no effect on mortality. Based on an unadjusted meta-analysis of data from 36 cases, Jansen et al. concluded that HBO might add therapeutic benefits in treating COVID-19 induced hypoxia as an adjunct to standard care [11].

The short existence of COVID-19 and of LCS notwithstanding, there are already a few reports about the successful application of HBO to post-COVID-19 syndrome. Bhaiat et al. published a case of successful HBO treatment of LCS in a former athlete who dramatically improved cognition deficits and cardiopulmonary function as documented by elaborate pre- and post-treatment workup, including spiro-ergometry and functional magnetic resonance imaging (MRI) [12].

The six LCS patients treated by Zant et al. suffered predominantly from muscle pain, joint pain, and dyspnea. Five out of six experienced improvements to pre-infection levels whilst the remaining patient reported significant relief [13].

Robbins et al. treated 10 consecutive patients in 10 sessions of HBO at 2.4 atmospheres over 12 days, focusing on fatigue and cognitive impairments. HBO yielded a statistically significant improvement of fatigue, global cognition, executive function, attention, information processing and verbal function [14].

Turova et al. performed a prospective, observational trial on HBO as an adjunctive measure in 45 patients participating in an outpatient rehabilitation program for LCS. They found the use of HBO beneficial in terms of improvement of functional and laboratory parameters [15].

Recently, in a large, prospectively randomized study, Zilberman et al. described the improvement of neuropsychological and neurocognitive symptoms in LCS [16] and the same group analyzed the neuro-structural background of the HBO action on LCS [17].

Based on the encouraging results mentioned above, we initiated an opportunistic and exploratory study on HBO in LCS focusing on both feasibility and outcome according to self-reporting by patients with symptoms from all three LCS clusters.

### 1.2. Hypothesis and Objectives

The overall hypothesis to be evaluated is that HBO is a safe and feasible treatment to alleviate symptoms associated with LCS.

The primary objective is to evaluate if HBO improves physical functioning, physical role, energy, emotional role, emotional well-being, social functioning, pain, general perception of health and limitation of activities.

The secondary objective is to evaluate if HBO has a beneficial impact on blood pressure, heart rate and peripheral oxygen saturation.

## 2. Materials and Methods

### 2.1. Study Design

This clinical, prospective, observational single-center pilot study was approved by the Local Institutional Review Board of the Medical University of Graz (No. 33-308 ex 20/21). Written informed consent was obtained from each patient before participation in the study. The study was conducted in accordance with the Declaration of Helsinki and according to good clinical practice.

### 2.2. Patient Characteristics

Seventy patients aged between 18 and 90 years with proven LCS were enrolled in the study between April 2021 and August 2022. All of them had had COVID-19 infection proven by a polymerase chain reaction (PCR)-test and an at least 3 months’ history of a minimum of two typical symptoms such as fatigue, dyspnea, dizziness, muscle weakness, insomnia, joint pain, myalgia or headache.

Patients with active malignancy, chest pathology incompatible with pressure changes such as pulmonary emphysema or moderate to severe asthma and pathological findings on electrocardiogram (ECG), spirometry, ear, or sinus pathology incompatible with pressure changes, pregnant or breast-feeding patients and those who had been administered HBO due to other reasons were excluded from the study. Patients unable to give informed consent and those with an active phase of COVID-19 infection were not included in the present study.

### 2.3. HBO Treatment

HBO treatment was carried out on an outpatient basis in a large walk-in, drive-in hyperbaric chamber. Each patient underwent a cycle of 10 subsequent HBO treatment sessions. HBO sessions were carried five times a week with a weekend break (two series of five compressions were performed). Each session lasted 75 min for a scheduled total time of 12 h and 30 min per patient. HBO was administered at a pressure of 2.2 atmospheres using medical oxygen. During compression the patients breathed 100% oxygen.

### 2.4. Patient Evaluation

Patient evaluation was carried out at three defined time points: immediately before HBO, immediately after the 10th HBO session and after 3 months.

These three evaluations were conducted in the same structured manner during the forenoon and consisted of the collection of both the patient’s circulation parameters and the data from self-reporting questionnaires about their health-related quality of life (HRQoL).

Among the patient´s circulation parameters, measurement of the blood pressure and the heart rate using a standardized electronic blood pressure meter was carried out. Measurement of the peripheral oxygen saturation was performed using a standardized pulse oximeter finger device.

With regard to their quality of life, the patients were asked to fill in the Short-Form-36 questionnaire (SF-36) and the visual analog scale (VAS).

The SF-36 survey is a widely used standardized questionnaire consisting of 36 self-reported items that are grouped into 8 dimensions [18]. These eight dimensions (physical functioning; physical role; energy; emotional role; emotional well-being; social functioning; pain; general perception of health) were used as main outcome measures in our exploratory analysis. In addition, the SF-36 questionnaire comprises a list of 38 everyday activities, where patients have to judge whether they were limited in performing these activities. The question proposed for the visual analogue scale was “How would you rank your present health and fitness by using this scale?”, where the scale extended across a distance of 100 mm. The mark set by the patient was measured with a ruler and recorded as mm from 0, where 0 indicated the worst case and 100 the best case [19,20,21].

The VAS has been widely used in medical research for several decades, especially for the measurement of pain. This numeric score describes the patients’ general perception of the severity of the disease on a 10-piece scale, with 10 indicating the highest severity [22].

### 2.5. Statistical Analysis

Descriptive statistics included mean, standard deviation, range and absolute and relative frequency where appropriate. For statistical analysis of pre- and post-treatment values, we used Student’s *t*-test for paired samples and—when there was no normal distribution—the Wilcoxon matched pairs signed rank test. Testing for normality was performed using the Shapiro–Wilk test. Since it is an exploratory study, we did not apply any correction for multiple comparisons. As far as multiple independent variables were concerned, however, we applied a multivariable statistical test. The evaluation was based on an intention-to-treat analysis.

Regarding power analysis using G*Power [23], a sample size of 45 cases is large enough to detect an effect size of 0.5 between pre- and post-treatment values with alpha = 0.05 and power (1-beta) = 0.95.

In addition, we used stepwise multivariable regression analysis. As independent parameters, we included age, sex, respiratory support during the acute COVID-19 episode, comorbidity of any type, diabetes, hypertension, body mass index and time between onset of the acute disease and the implementation of HBO therapy. We tested the relationship of these independent variables for each of the dependent variables. These were systolic and diastolic blood pressure, heart rate, O2 saturation, the eight dimensions of the SF-36 questionnaire, limitations in 38 different activities, and the results of the visual analogue scale. For each dependent variable, the difference between the values after HBO and the values before HBO were used. A negative difference indicates improvement, and a positive difference indicates worsening of the particular parameter. *p* < 0.05 was considered to indicate statistical significance.

## 3. Results

### 3.1. Study Population

**Figure 1:**
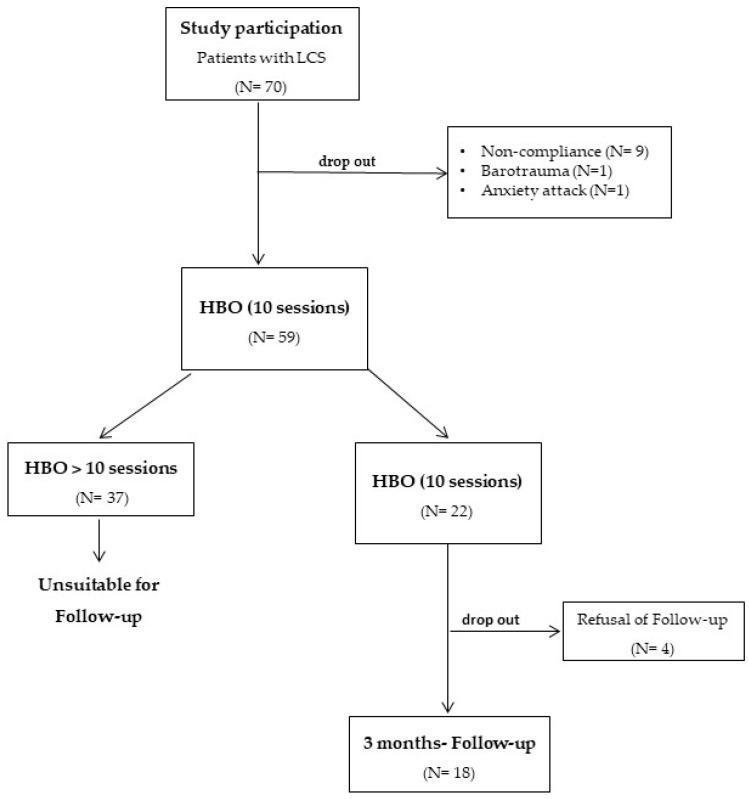
Study flow chart. Abbreviations: LCS: long COVID syndrome; HBO: hyperbaric oxygenation.

After the dropout of 11 patients (9 due to non-compliance, 1 because of barotrauma to the middle ear and 1 because of an anxiety attack during treatment) 59 patients (33 females, 26 males; mean age: 43.9 years; range: 23–74 years; median: 45.0) were evaluable for the study and had the planned number of HBO sessions. Thirty-seven patients wished to continue the HBO treatment beyond the 10th session due to subjective improvement of symptoms. They had a varying number of further HBO sessions and were excluded from the statistical evaluation beyond this time point. Out of the 22 remaining patients, 18 entered the final evaluation at 3 months, whilst 4 declined to show up for the investigation (Figure 1).

### 3.2. Severity of COVID-19 Infection

Fifty-three patients had had mild COVID-19 symptoms treated at home, and two had been hospitalized for medication and oxygen administration via a facemask. Four patients had required intensive care unit (ICU) treatment with intubation in one and tracheotomy in three of them. In stepwise multivariable regression analysis, the severity of the initial COVID-19 infection, as indicated by the requirement of oxygen or respiratory support measures, showed a more pronounced reduction of systolic blood pressure (t = −2.17, *p* = 0.03) and of pain (t = −2.76, *p* = 0.01) during the course of HBO therapy. The degree of improvement of all other parameters was not affected by the severity of the acute COVID-19 disease. The time between onset of acute COVID-19 and implementation of HBO therapy did not influence the degree of improvement of any of the outcome parameters.

### 3.3. Biometrical Data and Co-Morbidity

Mean body mass index (BMI) at the time of admission to the study was 25.3 (range: 18.6–38.2). Forty-one patients (69.4%) had some type of comorbidity with hypercholesterinemia (N = 21; 35.5%), found most frequently followed by hypertension (N = 20; 33.9%), obesity (N = 14; 23.7%) diabetes (N = 3; 5.0%) or coronary heart disease (N = 2; 3.3%). Twenty-five patients (42.3%) had further relevant preexisting disease such as allergic asthma, depression, disorders of the thyroid, or migraines. Neither age, sex nor co-morbidities had any statistically significant influence on the effect of HBO treatment measured by the outcome parameters. Only a high BMI slightly reduced the HBO treatment effect on the number of impaired activities (t = 2.04, *p* = 0.05). The two subgroups (those treated on schedule and those who continued HBO beyond 10 sessions) did not show any statistically significant differences concerning sex, age, biometrical data and co-morbidity.

### 3.4. Collective of Patients Finishing HBO Treatment (10 Sessions) without Follow-Up

Among these 59 patients, a slight, but statistically not significant decrease of the mean blood pressure was observed, whereas the mean heart rate decreased significantly (*p* = 0.03). Mean peripheral oxygen saturation remained nearly unchanged.

Physical functioning improved significantly (*p* < 0.001) after treatment and so did the physical role (*p* = 0.01). The same was true for social functioning, which improved significantly (*p* < 0.001). The limitation of activities according to SF-36 also decreased significantly after treatment (*p* < 0.001). Patients felt significant improvement also for energy (*p* < 0.001), emotional well-being (*p* < 0.001) and pain (*p* = 0.01). Improvement that failed to reach statistical level of significance was found for emotional role (*p* = 0.26) and general perception of health (*p* = 0.07).

The mean pre-therapeutic VAS score describing the patients´ general perception of the severity of their actual disease was 5.85 +/− 2.01 on a 10-piece scale, with 10 indicating the highest severity. After HBO, the score decreased significantly to 3.79 +/− 2.11 (*p* < 0.001).

The details are given in Table 1.

### 3.5. Collective of Patients Finishing HBO Treatment (10 Sessions) without Follow-Up

Among these 22 patients, the circulation parameters changed but without reaching statistical significance. The mean systolic blood pressure slightly decreased, whereas the mean diastolic blood pressure nearly remained unchanged. Mean heart rate and mean peripheral oxygen saturation slightly decreased.

Physical functioning (*p* < 0.001), energy (*p* = 0.02), social functioning (*p* = 0.02) and limitation of activities (*p* < 0.001) improved significantly after 10 sessions. All other parameters ascertained by the SF-36 survey also showed an improvement after HBO treatment but without statistical significance.

The mean VAS score reported pre-treatment decreased significantly from 5.5 to 3.3 (*p* < 0.001).

The details are presented in Table 2.

### 3.6. Collective of Patients Finishing HBO Treatment (10 Sessions) with Follow-Up

Among these 18 patients, mean systolic blood pressure, mean diastolic blood pressure and mean heart rate continued to decrease after 3 months compared to pre-HBO values. The mean peripheral oxygen saturation slightly increased, but none of these changes showed statistical significance at long-term follow-up.

Among the ascertained SF-36 parameters, physical functioning (*p* = 0.05), social functioning (*p* < 0.001) and limitation of activities (*p* = 0.02) improved even after 3 months as compared to their pre-HBO values, reaching statistical significance. The other SF-36-related parameters also showed an improvement after HBO treatment but without statistical significance.

Regarding the mean VAS, the change from the pre-treatment 5.2 to 2.6 post-treatment was still significant even after 3 months (*p* < 0.001).

The details are given in Table 2.

### 3.7. Collective of Patients Exceeding 10 HBO Sessions without Follow-Up

Among these 37 patients, the pre-treatment mean systolic blood pressure was slightly elevated and the mean diastolic pressure decreased slightly, but both without statistical significance. The mean heart rate decreased significantly (*p* = 0.04), whereas mean peripheral oxygen saturation remained nearly unchanged within the normal range.

Physical functioning improved significantly after 10 HBO sessions (*p* < 0.001). Physical role improved significantly (*p* = 0.05). The same was true for energy (*p* < 0.001), emotional well-being (*p* < 0.001), social functioning (*p* < 0.001) and limitation of activities (*p* < 0.001). All further SF-36 parameters also improved, yet without reaching statistical significance.

The mean VAS score improved statistically significantly from 6.06 +/− 1.84 to 4.08 +/− 1.9 (*p* < 0.001).

The details are given in Table 3.

### 3.8. Side Effects

Three patients experienced problems in pressurization of the middle ear, which were overcome by decongestant nose drops in one case and insertion of a vent tube in the eardrum in the second case. The third patient declined eardrum venting and had to discontinue the treatment. Another patient experienced an anxiety attack during treatment, which also led to discontinuation. There were neither acute nor prolonged side effects of HBO.

## 4. Discussion

This clinical prospective, observational pilot study demonstrates that HBO may provide a safe and feasible therapeutic tool for mitigation of LCS-related symptoms in both the short-term and the long-term follow-up. After 10 HBO treatment sessions, a statistically significant improvement in 80% of the ascertained items of the SF-36 survey together with a significant decrement of the VAS were obtained immediately. Even after 3 months, the statistically significant improvement in physical and social functioning and the reduction of limitations persisted. The subjective perception of the severity of disease mirrored by the VAS also remained significantly decreased.

HBO has been applied for decades for various indications requiring tissue repair in the broadest sense. They cover a wide range of acute and chronic diseases from ischemia-reperfusion injury to impaired wound healing, radiation-induced tissue damage and central nervous injury [24]. Since the turn of the millennium, the underlying mechanisms have gradually been elucidated. HBO affects various molecular pathways including transcription, vascular signaling and the response to oxidative stress. In addition, structural cellular components involved in angiogenesis, epithelization or collagen formation, cell-to-cell contacts, adhesion and transmigration are modified. The regulatory effects of HBO on apoptosis, autophagy, and cell death are further assets in regenerative processes. Additionally, HBO is a potent regulatory effector of inflammatory mechanisms [6].

There are numerous hypotheses about the causes of LCS. Although some findings indicate that LCS may result from prolonged organ damage mainly due to hypoxemia and coagulation disorders during the acute infection, specific processes following initial COVID-19 could trigger immune dysregulation, autoimmunity phenomena and endothelial dysfunction [5]. It is also plausible that neuronal damage, inflammation, or disturbance of transcription processes caused by occult viral persistence may play a role in this multisystem disease.

With regard to the neurophysiological characteristics of LCS, sub-optimal executive function associated with increased fatigue related to significantly reduced intracortical neurotransmission was confirmed [25]. These finding were corroborated by Ortelli and colleagues. They showed that patients with fatigue and cognitive disorders after COVID-19 infection presented altered excitability and neurotransmission with deficits in executive functions and attention [26]. Significant cerebral hypoperfusion affecting the frontal, parietal and temporal cortex leading to cognitive complaints could be detected as another causative in LCS [27]. These findings correlate with proven intracerebral hypometabolism verified by Positron emission tomography (PET). The affected cerebral regions ranged from the olfactory gyrus and connected (para-)limbic regions to the cerebellum and the brain steam and resulted in significantly increased functional complaints and clinical symptoms, i.e., hyposmia/anosmia, cognitive impairment, pain and insomnia [28].

In this context, HBO was shown to increase brain perfusion in the insula, hippocampus, putamen, and prefrontal and cingulate cortex. A further feature of HBO is its potential for stem-cell mobilization, neuro-regeneration and induction of neuroplasticity, which may mitigate neurological symptoms in LCS [16]. Based on these mechanisms, HBO can improve physical, neurocognitive and psychiatric symptoms related to LCS [29]. We could confirm these findings in the present study. After 10 HBO treatment sessions, we found immediate significant improvement of physical functioning, physical role, energy, emotional well-being, social functioning, and pain as well as significantly reduced limitation of activities as reported according to the SF-36 questionnaire. The subjective perception of the severity of disease mirrored by the VAS improved significantly, as displayed in Table 1.

Though patients with LCS may display objective pathological findings [29,30,31], many cases with pronounced symptoms show normal function tests, laboratory parameters and imaging. Authors have therefore suggested including LCS in the “unexplained post-infection syndromes” [32]. Accordingly, none of our patients had abnormal circulation parameters. Blood pressure, heart rate and peripheral oxygen saturation were within normal range, as displayed in Table 1, Table 2 and Table 3.

In consideration of this astonishing discrepancy between proven organ damage and inconspicuous function tests, the effectiveness of treatment in LCS is difficult to assess. Many patients are unable to describe their symptoms distinctly, though they do perceive them as severely debilitating. Fatigue is a common finding and so are memory issues, brain fog, attention disorder and sleep disturbance. Secondary anxiety and even psychiatric manifestations such as depression [33] contribute to subjective aggravation of symptoms. Because of the inherent vagueness of symptom descriptions by the patients, we solely relied on the self-reporting SF-36 survey [18,19,20,21] and on the 10-piece VAS [22] for evaluation of treatment effects. In this context, the methodology of the current study is in accordance with other authors who also used the SF-36 survey [16] and other standardized self-reporting questionnaires to assess the patient´s quality of life [13,14,17,21,29,33].

Of note, pain in LCS is refractory to most analgetic treatments [34] and to some degree resembles fibromyalgia, a central sensitization syndrome. The positive impact of HBO on pain, as shown in our study, resembles the effect of HBO on fibromyalgia as demonstrated by various authors [35,36]. As in our investigation, Zant et al. [13] and Zilberman et al. [16] reported significant pain relief in LCS. We could corroborate this findings in the current study. After HBO, a significant relief of pain according to the SF-36 survey and a significant decrease of the VAS could be confirmed, as shown in Table 1, Table 2 and Table 3. Due to these HBO-induced analgetic effects, 37/59 patients (63%) insisted on continuing the HBO treatment exceeding the scheduled 10 sessions, leaving only 18 patients evaluable for long-term assessment after 3 months, as shown in Figure 1.

To preclude differences in treatment response between the group that continued HBO and the one that adhered to the scheduled 10 sessions, we evaluated not only the total collective but also both groups separately. After 10 HBO treatments, the 37 patients who insisted on continuing HBO showed a statistically significant response for heart rate, and otherwise the same significant responses for SF-36 parameters and VAS as the total collective. By contrast, at the same time point, the 18 patients who finished HBO according to schedule displayed no statistically significant effect on heart rate, physical role, emotional well-being and pain, whereas the other positive effects of HBO as found in the total collective were present. Thus, the group that continued HBO had improvement in nine categories, whereas the “on-schedule” patients improved in only five categories.

The subjective impression of pronounced improvement that triggered the wish for continuation of treatment in the former group is confirmed by these data. Since there were no differences in the baseline criteria such as age, sex, biometrical data, severity of COVID-19 infection, or duration of symptoms, it is unclear why this group had a better response after 10 sessions.

In addition to the proven short-term effects induced by HBO in the present study, we were able to demonstrate the following long-term effects. In the subgroup of on-schedule patients we could show that the effect of HBO remained stable within 3 months following treatment. The statistically significant improvement in physical and social functioning and the reduction of limitations persisted. The subjective perception of the severity of disease mirrored by the VAS also remained significantly decreased. This proven long-term effect of HBO represents one strength of the current study in comparison to recent literature without evaluation of long-term data [14,16].

However, the second strength of the present study is the number of patients undergoing HBO treatment (N = 59). To the best of our knowledge, our study is the second-largest reported up to this time. In this context, Zilberman and colleagues investigated a larger collective consisting of 73 patients [16].

Despite applying only 10 HBO treatment sessions, our results resemble those of the prospectively randomized, sham control, double-blind study by Zilberman et al., who administered 40 HBO sessions per patient [16]. This enormous series of HBO sessions is the largest number documented in recent literature. Based on the assumption that an increasing number of HBO sessions could have a beneficial impact on the mitigation of LCS-related symptoms, the subgroup of 37 patients from the present study might confirm this suspicion. Feeling subjective improvement of their well-being during HBO, they insisted on continuing HBO treatment exceeding 10 sessions. However, according to our findings in comparison to matchable literature [14,16], we share the opinion that the optimal number of HBO sessions for maximal therapeutic effect has yet not be determined. However, Zilberman and co-workers were able to demonstrate significant improvement in global cognitive function, attention, executive function, the energy domain, sleep, psychiatric symptoms and pain. These findings are favorably in line with the results of the present study, which show a statistical significant improvement of clinical symptoms of the physical, the neurocognitive and the psychiatric areas, as documented in Table 1, Table 2 and Table 3. In contrast to our current study, the clinical features were mirrored by improvement in brain MRI perfusion and functional magnetic resonance changes in the respective areas. These findings suggest a change of the functional connectivity and organization of neural pathways by induction of neuroplasticity following HBO treatment [16,17].

Recently, the same group investigated the effect of HBO on left ventricular function in patients with LCS in a prospective, randomized study. Despite normal ejection fraction, almost half of the collective of 60 patients had reduced global longitudinal strain (GLS) at baseline. Following HBO, GLS increased significantly as a sign that HBO promotes myocardial recovery. This could at least in part explain the positive effect of HBO on physical function and energy [37], as we could confirm in the findings of the current study with significant improvements of both physical function and energy, as displayed in Table 1, Table 2 and Table 3.

However, our study has two limitations that have to be addressed: The most prominent feature was the prospective, observational study design without a control group. Due to technical issues (because of the lack of a gas blender), it is not possible to deliver hyperbaric sham treatment in our hyperbaric chamber. This is why we had to conduct the current study without a control group. Another shortcoming was the fact that about two thirds of the patients had wanted to continue HBO treatment beyond the planned 10 sessions after they had noticed a subjective improvement of their well-being during HBO. For this reason they were unfortunately unsuitable for the scheduled 3 months’ evaluation. In consequence, the latter is based on only 18 cases, as displayed in Figure 1.

## 5. Conclusions

In conclusion, though the pathogenesis of LCS is still unclear, and hence the specific mechanisms of HBO must remain speculative, HBO may provide a safe and feasible therapeutic tool for mitigation of LCS-related symptoms. Regarding the findings of the present clinical pilot study, we are able to conclude that after administration of HBO, physical functioning and both the physical and emotional role improved significantly and sustainably even during long-term follow-up. However, there is a strong need for further, prospectively randomized studies focusing on dose-finding, duration of HBO, elucidation of mechanisms and duration of the treatment effects.

## Figures and Tables

**Table 1:** Circulation parameters, peripheral oxygen saturation and data from self-assessment; pairwise comparison of pre- and post-treatment data. Total collective that finished 10 HBO treatment sessions.

	Pre-HBO (N = 59)	Post-HBO (N = 59)	*p* Compared with Pre
Mean systolic blood pressure (mm Hg)	131.9 +/− 20.0	130.6 +/− 15.8	0.46
Mean diastolic blood pressure (mm Hg)	81.3 +/− 12.1	79.3 +/− 10.2	0.09
Mean heart rate (bpm)	80.3 +/− 12.9	76.8 +/− 11.4	**0.03**
Peripheral oxygen saturation (%)	96.0 +/− 1.5	96.2 +/− 1.5	0.35
Physical functioning	43.9 +/− 24.6	52.4 +/− 24.6	**<0.001**
Physical role	10+/− 27.4	16.8 +/− 30.4	**0.01 (0.02)**
Energy	22.3 +/− 20.2	30.4 +/− 20.4	**<0.001 (<0.001)**
Emotional role	45.0 +/− 48.1	51.2 +/− 46.5	0.26
Emotional well-being	54.0 +/− 18.5	64.0 +/− 18	**<0.001**
Social functioning	32.6 +/− 26.6	43.7 +/− 25.9	**<0.001**
Pain	42.7 +/− 25.0	50.1 +/− 22.1	**0.01**
General perception of health	39.5 +/− 16.8	42.9 +/− 16.3	0.07
Limitation of activities	12.8 +/− 5.5	9.4 +/− 7.9	**<0.001**
VAS score	5.85 +/− 2.01	3.79 +/− 2.11	**<0.001**

*t*-test for paired samples; *p* values in parenthesis were calculated using the Wilcoxon matched pairs signed rank test. Abbreviations: HBO: hyperbaric oxygenation; mmHg: millimeters of mercury; bpm: beats per minute; %: percent; VAS: visual analog scale.

**Table 2:** Circulation parameters, peripheral oxygen saturation and data from self-assessment; pairwise comparison of pre-treatment data, post-treatment data after 10 HBO sessions and data after 3 months.

	Pre-HBO (N = 22)	Post-HBO (N = 22)	*p* Compared with Pre	Pre-HBO (N = 18)	After 3 Months (N = 18)	*p* Compared with Pre
Mean systolic blood pressure (mm Hg)	135.3 +/− 23.8	131.5 +/− 15.6	0.25	141.4 +/− 24.1	131.3 +/− 11.8	0.06
Mean diastolic blood pressure (mm Hg)	82.8 +/− 10.9	82.5 +/− 10.3	0.79	86.1 +/− 10.9	81.4 +/− 8.9	0.09
Mean heart rate (bpm)	82.2 +/− 12.7	80.7 +/− 12.4	0.47	81.9 +/− 13.0	80.1 +/− 10.4	0.45
Peripheral oxygen saturation (%)	96.0 +/− 1.4	95.7 +/− 1.5	0.38	96.1 +/− 1.7	96.7 +/− 1.7	0.41
Physical functioning	46.8 +/− 25.6	57.3 +/− 26.4	**<0.001**	44.1 +/− 26.7	58.3 +/− 24.7	**<0.001**
Physical role	14.4 +/− 32.6	21.0 +/− 35.6	0.14 (0.25)	16.1 +/− 34.7	30.9 +/− 41.9	0.07 (0.13)
Energy	26.8 +/− 25.1	36.5 +/− 20.9	**0.02** **(0.01)**	30.8 +/− 26.0	38.0 +/− 25.9	0.15 (0.13)
Emotional role	41.6 +/− 48.2	50 +/− 48.9	0.06	42.6 +/− 49.6	50.0 +/− 46.1	0.16
Emotional well-being	54.1 +/− 22.4	61.4 +/− 21.1	0.07	58.3 +/− 46.7	64.4 +/− 24.5	0.09
Social functioning	38.1 +/− 31.2	48.1 +/− 29.3	**0.02**	39.6 +/− 32.7	60.4 +/− 32.4	**<0.001**
Pain	49.3 +/− 28.1	53.5 +/− 24.0	0.42	52.9 +/− 28.2	58.2 +/− 30.2	0.17
General perception of health	38.3 +/− 20.7	42.7 +/− 20.3	0.17	43.2 +/− 19.6	46.2 +/− 23.9	0.49
Limitation of activities	13.0 +/− 6.9	9.2 +/− 5.7	**<0.001**	12.2 +/− 7.1	9.2 +/− 6.8	**0.02**
VAS score	5.5 +/− 2.3	3.3 +/− 2.4	**<0.001**	5.2 +/− 2.3	2.6 +/− 1.9	**<0.001**

*t*-test for paired values; *p*-values in parenthesis were calculated using the Wilcoxon matched pairs signed rank test. Abbreviations: HBO: hyperbaric oxygenation; mmHg: millimeters of mercury; bpm: beats per minute; %: percent; VAS: visual analog scale.

**Table 3:** Circulation parameters, peripheral oxygen saturation and data from self-assessment; pairwise comparison of pre- and post-treatment data. Collective non-eligible for 3-months evaluation due to continuation of HBO beyond 10 treatment sessions.

	Pre-HBO (N = 37)	Post-HBO (N = 37)	*p* Compared with Pre
Mean systolic blood pressure (mm Hg)	129.9 +/− 17.3	130.1 +/− 16.1	0.94
Mean diastolic blood pressure (mm Hg)	80.4 +/− 12.8	77.4 +/− 9.8	0.08
Mean heart rate (bpm)	79.2 +/− 13.1	74.4 +/− 10.2	**0.04**
Peripheral oxygen saturation (%)	96.1 +/− 1.5	96.6 +/− 1.5	0.09
Physical functioning	42.2 +/− 24.2	49.5 +/− 23.3	**<0.001**
Physical role	7.6 +/− 24.5	14.6 +/− 27.6	**0.05** (0.05)
Energy	19.8 +/− 16.8	27.1 +/− 19.7	**<0.001** **(<0.001)**
Emotional role	47.0 +/− 48.6	51.9 +/− 45.8	0.56
Emotional well-being	54.0 +/− 16.3	65.6 +/− 15.9	**<0.001**
Social functioning	29.4 +/− 23.4	41.2 +/− 23.7	**<0.001**
Pain	38.9 +/− 22.6	48.2 +/− 21.1	**<0.001**
General perception of health	40.1 +/− 14.7	43 +/− 14.1	0.22
Limitation of activities	12.6 +/− 4.6	9.6 +/− 5.7	**<0.001**
VAS score	6.06 +/− 1.84	4.08 +/− 1.9	**<0.001**

*t*-test for paired values; *p*-values in parenthesis were calculated using the Wilcoxon matched pairs signed rank test. Abbreviations: HBO: hyperbaric oxygenation; mmHg: millimeters of mercury; bpm: beats per minute; %: percent; VAS: visual analog scale.

## Data Availability

The data presented in this study are available upon request from the corresponding author.
